# Hydrometeorological shocks to infectious-disease systems: lessons from Pakistan's 2025 floods

**DOI:** 10.3389/fpubh.2025.1697850

**Published:** 2025-12-15

**Authors:** Jibran Ikram, Kanza Farhan, Khushbakht Baloch, Syeda Nashrah Ayaz, Muhammad Burhan Tariq, Izere Salomon

**Affiliations:** 1 Department of Cardiovascular Medicine, Cleveland Clinic Tomsich Family, Cleveland, OH, United States; 2 Sindh Medical College, Jinnah Sindh Medical University, Karachi, Pakistan; 3 College of Medicine and Health Sciences, University of Rwanda, Kigali, Rwanda

**Keywords:** flood, disaster and risk management, disaster surveillance, infectious disease, climate change

## 1 Introduction

The 2025 monsoon flood in Pakistan led to catastrophic mortality, mass displacement, and large-scale destruction of water, sanitation, and hygiene (WASH) systems. These floods have triggered a severe health crisis as millions remain in overcrowded shelters that lack basic health facilities and have limited clinical surge. Due to WASH collapse, uncontrolled vector proliferation, shelter crowding, and fragile supply chains, such hydrometeorological extremes predictably escalate into infectious disease emergencies (1). It is evident from clearly connecting these occurrences to flood patterns caused by climate change that extreme weather now directly increases the spread of infectious diseases, resulting in a cascading public health calamity. To avert a subsequent calamity, robust vector control, restoration of WASH, proactive testing, and case management that is cognizant of antimicrobial resistance (AMR) are imperative for both Pakistan and global health security. Therefore in addition to the initial devastation caused by the storm, waterborne and foodborne infections are major concerns (2).

## 2 The cascading post-disaster infectious disease crisis

Cholera and enteric fever require immediate action as they severely constrain treatment capacity. Leptospirosis and Hepatitis A and E pose a heavy risk and require urgent preparedness as they are historically recorded epidemics after floods. Meanwhile, stagnant water provides a breeding source for mosquitoes, thereby causing a surge in vector-borne diseases (3). Shelter overcrowding has amplified acute respiratory infections, scabies, skin infections, and secondary wound infections, while tetanus risk is fueled in trauma cases due to a lack of timely prophylaxis. Polio resurgence is also a major threat, fueled by hurdles in immunization campaigns due to severe flooding, as Pakistan remains one of the only two polio-endemic countries (4).

## 3 Multi-layered intervention strategies and recommendations

### 3.1 Strengthening surveillance and early warning systems

A multipronged, disciplined system for syndromic surveillance and vector control should be administered across all the affected districts. In order to safeguard the population of Pakistan and to protect global health safety, rapid diagnostic tests (RDTs) should be coupled with surveillance systems, especially for cholera, dengue, and malaria (4, 5). Internally displaced person camps and settlements should be assessed for systematic symptoms of watery diarrhea, undifferentiated febrile illness, febrile jaundice, hemorrhagic fever, and neurological syndromes. For further validation of the rapid testing, provincial laboratory networks should maintain confirmatory microbiology assessments. Surveillance outcomes should adhere to the guidelines provided by Pakistan's National Institute of Health Integrated Disease Surveillance and Response (IDSR), including recommended minimal datasets that are shared regularly through weekly public bulletins (6).

### 3.2 Frontline clinical management and AMR response

In post-flood Pakistan, clinical management requires reinforced, agile stewardship fused with conventional field triage (7). Mobile antimicrobial stewardship teams, armed with simple traffic-light antibiotic decision charts, must deploy with every field clinic to shape frontline antibiotic rules. Community sentinel nodes must host rapid, multiplex tests for cholera, dengue, malaria, and typhoid, catalyzing rapid differential diagnostics (8).

For acute watery diarrhea and cholera, rehydration supply pathways and intravenous reuse remain the core response, with isolation capacity added. Avoid generic fluoroquinolone for typhoid; instead, patients should step to resistance-informed, higher-tier treatment pathways, layered with culture-based vigil for XDR tracking.

For dengue, algorithm adherence is paramount; guard against unnecessary antibiotics and antiphlogistic misuse, and restrict platelet transfusions to clear clinical indications to avoid wastage. Include malaria test-and-treat tactics insisting on appropriate artemisinin-based combination therapy, in tandem with G6PD screening before primaquine/tafenoquine in *Plasmodium vivax* (7, 8). Wound standards stress early debridement and tetanus for a time. To facilitate prompt clinical decision-making, field clinics and laboratories should promote quick, two-way communication. Beyond single clinical measures, water hygiene and availability, and biodiversity management must rise to a clinical role (7–9).

### 3.3 Integrated WASH and vector control

Emergency chlorination, targeted at both the source and household levels, combined with turbidity control, elevated latrines, and managed washing zones, continues to disrupt waterborne transmission (9). A broad circle of residents must maintain container hygiene, and regular volunteer sweeps curb algal breeding hotspots. When drains and latrines overflow with turbid water, rapid repair nets the flow back. Over the same stretch, vector suppression adds control: short-lifecycle larviciding, indoor residual spraying at temporary sites, and community pack-outs of nets, mosquito repellents, and window screens (9).

### 3.4 Building systemic resilience

To complete the protective package, immunization protocols must include oral cholera vaccines where available, universal tetanus boosters for newly wounded, and dose-targeting for hepatitis among dense relocation settings, all layered atop WASH, behavioral change, and environmental safeguards (10). Climate adaptation funds and loss-and-damage financing should be directed at improving laboratory capacity, resilient water systems, and mobile health outreach, with equity weighting focused on children, pregnant individuals, recent displacees, and inhabitants of peripheral sites.

## 4 Conclusion

Pakistan's recent flood starkly illustrates how shifting climate patterns amplify arboviral risks, notably dengue, positioning the country as an instructive prototype for neighboring states and demanding long-term multi-sectoral solidarity (10). The anticipated monsoon inundations of 2025 now emerge as both a sentinel event and an empowering reference point, one that the international infectious-disease fraternity must scrutinize to advance preparedness agendas for global safety and wellbeing.
